# Tubal Ectopic Pregnancy With Grossly Visible Intact Embryonic Structures: A Case Image

**DOI:** 10.1002/ccr3.73731

**Published:** 2026-10-12

**Authors:** Mojgan Akbarzadeh‐Jahromi, Neda Soleimani, Sahand Mohammadzadeh

**Affiliations:** ^1^ Department of Pathology, Shiraz Medical School Shiraz University of Medical Sciences Shiraz Iran

**Keywords:** ectopic pregnancy, fallopian tube, pregnancy, unruptured tubal pregnancies

## Abstract

An unruptured tubal ectopic pregnancy containing intact embryonic structures is a rare and life‐threatening condition demanding prompt diagnosis. The educational value of this report lies in its high‐quality gross image demonstrating intact embryonic structures within an unruptured tubal ectopic pregnancy.

## Key Clinical Message

Advanced unruptured ectopic pregnancy can rarely present with a preserved embryo within the fallopian tube. Early diagnosis is essential to prevent tubal rupture and life‐threatening hemorrhage.

Advanced unruptured tubal ectopic pregnancy with a visible embryo is uncommon. It represents a life‐threatening condition, which can be missed easily in the absence of classic symptoms of vaginal bleeding, abdominal pain, and amenorrhea [1].

A 41‐year‐old woman referred with lower abdominal pain and mild vaginal bleeding for 2 days. She had a history of amenorrhea for 7 weeks. Her past medical history included hypertension, for which she was receiving treatment. She denied any history of previous pregnancy and pelvic inflammatory diseases. Physical examination showed abdominal tenderness in the left lower quadrant with normal vital signs. Vaginal examination revealed cervical motion tenderness and a left adnexal lesion. Laboratory tests showed elevated βHCG titer (1240 mIU/mL). Pelvic ultrasonography showed no evidence of an intrauterine gestational sac and confirmed the presence of an adnexal lesion, suggestive of tubal ectopic pregnancy with a live embryo. Considering the presence of fetal cardiac activity and the treating team's clinical judgment, salpingectomy was performed. The pathology specimen was an intact, dilated fallopian tube measuring 6.5 × 3.8 cm. A longitudinal cut section shows an intact embryo with well‐preserved external morphology (Figure 1). Microscopic evaluation confirmed the presence of chorionic villi and trophoblastic tissue within the fallopian tube, consistent with tubal ectopic pregnancy.

**FIGURE 1:**
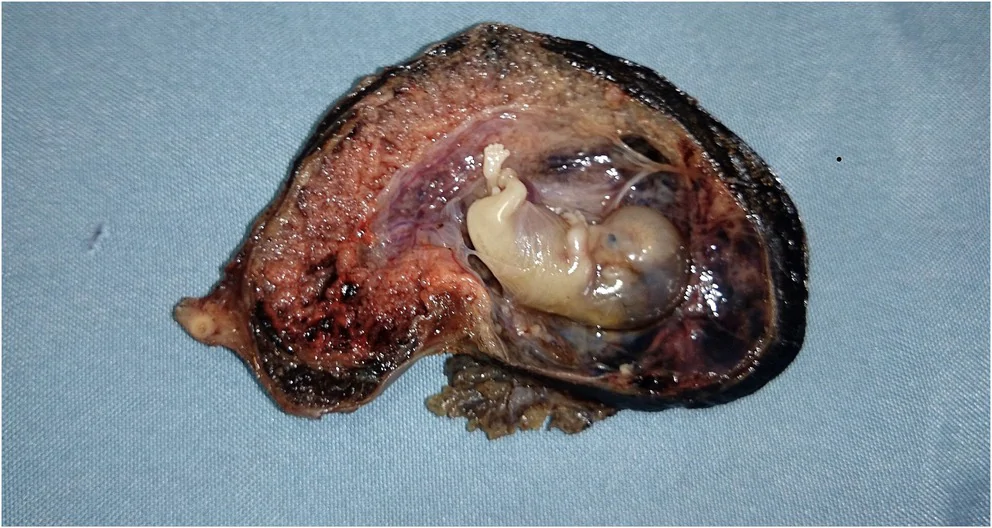
Longitudinally opened fallopian tube showing an intact embryo inside a gestational sac.

Ectopic pregnancy is one of the most important causes of morbidity during the first trimester of pregnancy. Most ectopic pregnancies occur within the fallopian tube, particularly the ampullary portion, and delayed diagnosis may result in tubal rupture and severe intra‐abdominal bleeding [2].

Advanced unruptured ectopic pregnancy with a grossly preserved embryo is uncommon. This image highlights the remarkable distensibility of the fallopian tube, allowing growth and preservation of an intact embryo. Although similar cases have been reported, the educational value of this report lies in its high‐quality gross image demonstrating intact embryonic structures.

Management of ectopic pregnancy depends on several factors, including clinical stability, β‐hCG level, imaging findings, fetal cardiac activity, and overall clinical judgment [3]. This case also highlights that serum β‐hCG concentration may not always correlate with the degree of embryonic development. Despite a relatively low β‐hCG level, the presence of fetal cardiac activity, together with the well‐developed embryonic morphology, favored surgical management in this case because these features are associated with a lower likelihood of successful medical treatment.

## Author Contributions

**Sahand Mohammadzadeh:** data curation, investigation, writing – review and editing. **Mojgan Akbarzadeh‐Jahromi:** conceptualization, supervision. **Neda Soleimani:** conceptualization, methodology, writing – review and editing, resources, writing – original draft.

## Funding

The authors have nothing to report.

## Ethics Statement

The study was conducted in accordance with the World Medical Association Declaration of Helsinki.

## Consent

The patient has given her written informed consent to publish the case (including the publication of images obtained). Written informed consent was obtained from the patient to publish this report in accordance with the journal's patient consent policy.

## Conflicts of Interest

The authors declare no conflicts of interest.

## Data Availability

Research data are not shared.
